# Exploring Mental Health Consumer Perceptions and Experiences of Goal‐Setting With the Physical Health Nurse Consultant

**DOI:** 10.1111/inm.70114

**Published:** 2025-08-05

**Authors:** Tracy Tabvuma, Robert Stanton, Ya‐Ling Huang, Brenda Happell

**Affiliations:** ^1^ Faculty of Health Southern Cross University Lismore New South Wales Australia; ^2^ School of Health, Medical and Applied Sciences Central Queensland University Rockhampton Queensland Australia; ^3^ School of Nursing and Midwifery University of Southern Queensland Ipswich Queensland Australia

**Keywords:** goal‐setting theory, health behaviour changes, mental health nurse, physical health, qualitative research

## Abstract

Despite the implementation of physical health policies, research and interventions, people diagnosed with mental health conditions (referred to as consumers) continue to experience increased morbidity and mortality compared with the general population. Underpinning this disparity, systemic and personal barriers continue to impede consumers' abilities, commitment and resourcing towards health behaviour changes. Evidence suggests appropriately skilled, interpersonally capable and empowered healthcare professionals like Physical Health Nurse Consultants can deliver person‐centred physical health care aligning with goal‐setting theory. However, little to no research focuses on the application of goal‐setting theory regarding physical health interventions for consumers. This qualitative exploratory study seeks to explore consumers' views and experiences using goal‐setting to co‐develop and implement their personalised health goals. Between November 2020 and April 2021, fourteen consenting consumers participated in 30‐to‐60‐min semi‐structured individual interviews that were transcribed and thematically analysed. Three themes identified from the data reflect consumers' positive experience of health goal‐setting with the PHNC. The themes communicate: (i) the process of goal‐setting, (ii) techniques, barriers and facilitators to implementing and sustaining, and (iii) impact of health goals. Consumers perceived collaborative care‐planning processes aligning with goal‐setting theory facilitated co‐development and implementation of varying health goals and goal types. Barriers to consumers' health goal attainment were effectively mitigated by the PHNC who applied elements of goal‐setting theory to increase congruency with their physical and mental capacity and commitment. Subsequently, consumers indicated positive impacts on several health domains indicating the value of the PHNC in supporting behaviour change and directing future research regarding consumer physical health interventions to underpin behaviour change theories and measure both clinical and personal outcomes.

## 1 Introduction

Mental health consumers, particularly those diagnosed with psychosis, continue to experience higher morbidity and mortality rates compared to the general population despite the implementation of physical health interventions and policies (Chan et al. 2023; O'Connor et al. 2023). This underscores the need for interventions addressing both systemic and personal barriers hindering consumers' ability to commit to and resource health behaviour change. Physical Health Nurse Consultants (PHNC) are appropriately skilled and empowered specialist mental health nurses who can deliver person‐centred physical health care (Happell et al. 2023; Tabvuma et al. 2024) that aligns with goal‐setting theory. Limited research explores the application of goal‐setting theory within the context of physical health interventions for consumers. The PHNC applied goal‐setting theory, alongside various behaviour change theories, in the delivery and coordination of physical healthcare for consumers by assessing and recognising their ability, commitment, and resource capacity. Consumers view this role as crucial to co‐developing and implementing their personalised health goals and effective support to mitigate barriers to their health goal attainment (Tabvuma, Stanton, and Happell 2022).

## 2 Background

Goal‐setting is widely practised in healthcare settings to facilitate health behaviour changes for people diagnosed with mental health conditions (hereon referred to as consumers) (Swann et al. 2021). Within healthcare settings, goal‐setting processes typically involve healthcare professionals facilitating structured conversations with consumers to establish, plan and implement health goals (Bailey 2019; Ogbeiwi 2021). In this paper, health goals refer to goals co‐developed by consumers to improve their physical health.

Goal‐setting theory enables the prediction, explanation and influence on an individual's performance (Bailey 2019; Locke and Latham 2019). In this paper, performance refers to an individual's ability and process of goal attainment, while performance feedback denotes evaluation of progress towards achieving the goal (Swann et al. 2021; Bailey 2019). Goal characteristics are classified as either learning‐ or performance‐based goal types depending on the objective (Locke and Latham 2019; Swann et al. 2021). Both characteristics require specific and challenging goals to be set. Goal specificity stimulates individual choice to take action and, combined with difficulty level, motivates effort from the individual (Locke and Latham 2019; Swann et al. 2021). Learning‐based goals are akin to process outcomes where the focus is to strategise an approach to implementing desired health behaviour changes, such as exploring a suitable time for a walk. Performance‐based goals focus on specific task outcomes, such as walking 30 min a day (Bailey 2019; Swann et al. 2021). These specific and challenging goals improve individuals' performances when mediated by four tenets: recognition of the individual's ability, commitment to the goal, access to resources, and feedback on performance (Bailey 2019; Ogbeiwi 2021; Swann et al. 2021).

A recent systematic review and meta‐analysis investigated the effectiveness of physical activity interventions delivered or prompted by healthcare professionals in primary care settings. Analysis showed a significant increase in self‐reported moderate to vigorous intensity physical activity (MVPA) of 14 min per week in the intervention group compared to the usual care group (Kettle et al. 2022). Whilst these results may appear modest, the increased MVPA is associated with an 11% decrease in mortality risk (Ekelund et al. 2019; Kettle et al. 2022). Findings from a more recent systematic review and meta‐analysis indicated that using multiple behaviour change techniques (BCTs) in combination is recommended for effective smoking cessation care (Mersha et al. 2023). These BCTs included health consequences education, problem solving, behaviour exposure, avoidance or reduction, and goal‐setting (Mersha et al. 2023). It is noted that studies included in the reviews leading to positive health behaviour changes were underpinned by behaviour change principles. Evidently, robust goal‐setting with multifaceted approaches and mediated by four tenets of goal‐setting theory facilitates health behaviour changes.

For consumers, mortality disparity between 15 and 30 years continues to exist despite implementation of numerous physical health policies, research, and interventions (Chan et al. 2023; Fiorillo and Sartorius 2021; Roberts et al. 2022). This disparity is underpinned by systemic and personal barriers such as inadequate physical health care, adverse drug reactions from antipsychotic medication, and negative impacts of mental illness (Bailey et al. 2020; Bort‐Roig et al. 2019; Department of Health 2017; Hassan et al. 2020). Efforts to address systemic barriers are evident in international and national policies (Department of Health 2017; O'Connor et al. 2023), physical health screening tools and multidisciplinary interventions (Lamontagne‐Godwin et al. 2018; Curtis et al. 2024), adverse drug side‐effect management (Curtis et al. 2012, 2024) and nurse‐led interventions (Happell et al. 2023). Despite these efforts, evidence suggests reducing the mortality gap has been hindered by inadequate healthcare professional physical healthcare knowledge and skills, gaps in evidence synthesis and inadequate implementation exacerbated by the effects of stigma and discrimination (O'Connor et al. 2023; Hassan et al. 2020).

Physical health interventions seeking to reduce mortality disparity typically involve lifestyle behaviour changes targeting consumers' diet and physical activity (Bradley et al. 2022; Bailey et al. 2020). However, these interventions report varying degrees of success, which can be attributed to the additional and complex barriers consumers' experience (Bradley et al. 2022; Kettle et al. 2022). In the context of health behaviour change, personal barriers limit consumers' ability and commitment to implement and sustain behavioural changes (Swann et al. 2021). Failing to assess consumers' capacity to attain, commit, and resource specific health goals potentially results in poor performance (Swann et al. 2021; Locke and Latham 2019). Considering these additional consumer complexities, examining behavioural change approaches used by healthcare professionals is crucial to establishing, implementing, and sustaining consumer health goals.

Appropriately skilled, interpersonally capable and empowered healthcare professionals like Physical Health Nurse Consultants (PHNC) are necessary to deliver person‐centred physical health care aligning with goal‐setting theory (Tabvuma, Stanton, and Happell 2022; Tabvuma et al. 2024). The PHNC is a specialist mental health nurse trained to deliver, coordinate, and integrate physical and mental healthcare (Happell et al. 2023; Tabvuma et al. 2024). The role has previously been recognised as facilitating goal‐setting with consumers; however, early work focused on therapeutic partnerships between the PHNC and consumers (Tabvuma, Stanton, and Happell 2022). Similarly, other studies regarding physical health interventions seldom report consumer experiences with goal‐setting (Bradley et al. 2022; Kettle et al. 2022). Limited research focuses exclusively on exploring elements of goal‐setting theory application within the context of specific physical health interventions for consumers.

## 3 Aim

This study seeks to explore consumers' views and experiences using goal‐setting to co‐develop and implement their personalised health goals. Elements of goal‐setting theory application within the context of consumers working with the PHNC will be investigated. Understanding consumer perceptions and experiences with goal‐setting in relation to physical health intervention provides insights for approaches to effectively co‐develop, implement and sustain health goals. To our knowledge, this may be the first study exploring consumer perceptions and experiences of goal‐setting and identifying elements of goal‐setting theory when co‐developing and implementing health goals with the PHNC.

## 4 Materials and Methods

### 4.1 Study Design

A qualitative exploratory design was used to enable the exploration of phenomena that are yet to be understood (Stebbins 2001). Consumer views and experiences with goal‐setting and investigation of goal‐setting theory application by the PHNC have not yet been researched. Participants openly sharing their insights will provide a focus for future research and aid with the application of goal‐setting theory in physical health care for mental health consumers. This manuscript adheres to the COREQ (COnsolidated criteria for Reporting Qualitative research) guidelines for reporting qualitative research (Tong et al. 2007).

### 4.2 Setting

Data collection was conducted between November 2020 and April 2021 at a large public Community Mental Health Service in the Australian Capital Territory (ACT). Community Mental Health Services such as assertive care, day services and outpatient programmes are delivered to adults aged 18–65 years diagnosed with varying mental health conditions.

### 4.3 Physical Health Nurse Consultant Role

The PHNC is a specialist mental health nursing role developed to deliver and coordinate physical health care for consumers diagnosed with psychosis (Happell et al. 2018). The PHNC worked with consumers to facilitate health goal‐setting, planning and implementation (Happell et al. 2018). Each consumer was provided tailored health advice and coaching to mitigate personal barriers and achieve self‐identified health goals. The PHNC facilitated holistic physical healthcare by referring consumers, when required, to allied health services such as dietetics or exercise physiology.

### 4.4 Study Population

A convenience and purposive sampling approach was used. Consumers who were adult English speakers diagnosed with psychosis and working with the PHNC were recruited. This is a common and efficient sampling approach for exploratory research involving recruiting accessible and consenting participants (Braun and Clarke 2021).

### 4.5 Ethics

The study adhered to the National Statement on Ethical Conduct in Human Research (Commonwealth of Australia 2018). Ethical approval was obtained from the ACT Health Human Research and Ethics Committee (approval number 2020/ETH.00081) prior to commencement. Prospective participants received Participant Information Statements explaining the purpose of the interview and how data would be collected, used and stored. Demographic characteristics were not collected due to the potential risk for inadvertent reidentification in accordance with the study's ethics approval. Participant identity remained confidential in the data analysis and reporting phase.

### 4.6 Recruitment

Consumers verbally consenting to an invitation into the study from healthcare staff had their contact details forwarded to the researcher. Potential participants were contacted by the lead author, a female credentialed mental health nurse with no prior relationship with participants. The independence of the researcher minimised bias. Participants were recruited into the study, provided additional information for deliberation, and assessed for their capacity to provide consent in accordance with the approved protocol. Consenting participants were sent a Participant Information Sheet and written informed consent form.

### 4.7 Procedure

Between November 2020 and April 2021, fourteen consenting consumers participated in 30‐to‐60‐min semi‐structured individual interviews (Table 1) conducted by the lead female author. Data saturation was reached when no new themes were identified and during the concurrent data analysis (Vasileiou et al. 2018). This occurred by the twelfth interview. During the interview period, the Australian government recommended physical distancing to manage COVID‐19 resulting in interviews being conducted via telephone or videoconferencing and audio recorded for transcription. Informed consent was obtained from all participants prior to the interview, demonstrating their agency to share their views and experiences. Consumers participating in videoconference interviews had the option to turn off their cameras. All participants were offered the choice to review their transcript. Participants were reimbursed $75 for their participation, aligning with the National Mental Health Commission ‘Paid Participation Policy’ guidelines (National Mental Health Commission 2020).

**TABLE 1:** Semi‐structured interview guide.

Please tell me about your experience with the physical health care nurse?
2 In what ways, if any has your physical health changed since seeing the physical healh nurse?
3 Can you tell me, what were the things that the physical health care nurse did that helped you the most?
4 What were the things that were less helpful?
5 What else could the physical health care nurse have done to improve your experience?
6 How easy was it to access the physical health care nurse/centre?
7 Is there anything else about this experience you think is important for me to know?

### 4.8 Analysis

Audio‐recordings were transcribed verbatim. Reflexive thematic analysis was conducted based on the 6‐step Braun and Clarke (2022) framework. The reflexive thematic analysis framework enables data organisation, pattern identification, analysis and reporting (Hassan et al. 2020). The initial phase of analysis involved researchers repeatedly reading transcripts for familiarity with the data. The following stage included using NVIVO 14 (QSR International, Burlington, MA) to identify and label specific areas of content into codes. These codes were collated, reviewed, and related back to the topic and searched for similarities. Next, similar codes were grouped, forming the basis of provisional themes. A conceptual map encompassing the tentative themes was titled and organised to broadly reflect key information from the data. Researchers reviewed the thematic structure, discussed interpretation discrepancies and refined themes to ensure accuracy and reflect the consensus view. Final themes were verified and defined by the research team for reporting in academic outputs (Braun and Clarke 2022).

### 4.9 Trustworthiness

Investigator triangulation and reflexivity were used to ensure rigour and limit bias (Carter et al. 2014). The research team, with PhD qualifications, backgrounds in nursing and exercise physiology, and qualitative research, separately thematically analysed and interpreted the data. The reflexivity process involved the research team reflecting on and challenging their interpretations of data, assumptions, and opinions about the research topic and resolving discrepancies until consensus was reached (Braun and Clarke 2022). Multiple perspectives and continual reflections about a phenomenon and interpretation of findings strengthen the trustworthiness and credibility of data (Carter et al. 2014; Braun and Clarke 2022). Participants had the opportunity to review and edit their transcript to accurately reflect their experiences and opinions.

## 5 Results

At the time of data collection, fourteen participants interviewed were accessing the community mental health service and reported previous experiences of hospitalisation for their mental health condition.

Three themes identified from the data reflect participants' positive experience of health goal‐setting with the PHNC. The themes convey: (i) the process of goal‐setting, (ii) techniques, barriers and facilitators to implementing and sustaining, and (iii) impact of health goals (Table 2).

**TABLE 2:** Themes and sub‐themes related to participants' positive experiences of health goal‐setting with PHNC.

Themes	Subthemes
The process of goal‐setting	Approaches used by the PHNC to co‐develop health goals
	Types of health goals co‐developed
Techniques, barriers and facilitators to implementing and sustaining health goals	Techniques for implementing health goals
	Barriers
	Facilitators
Impact of health goals	Impact of health goals

### 5.1 Theme 1: The Process of Goal‐Setting

Participants described positive goal‐setting involving co‐development of consumer‐defined health goals with the PHNC guiding the process as described in the following sub‐themes: approaches used by PHNC to co‐develop health goals and types of health goals co‐developed.

#### 5.1.1 Subtheme 1: Approaches Used by PHNC to Co‐Develop Health Goals

Participants highlighted their therapeutic partnership with the PHNC as a catalyst for health goal development and implementation. Therapeutic partnerships were strengthened by perceived PHNC attributes of being caring and empathising with participants' mental and physical health capacity and experiences.

There's people [PHNC] there that care…You can give up yourself but they're [PHNC] not giving up on you. (Participant 6)

Most participants described an empowering, conversational and professional approach to setting health goals. The PHNC explored their capacity, commitment and resources for health behaviour change. Participants described how the PHNC prioritised their autonomy and choice, and offered performance feedback, support and guidance throughout their journey.

There wasn't any pressure…they really extracted the goals from me, rather than suggesting goals. That was really important. It's like an empowering process…Having me identify those goals really puts the ball in my park—about what I want to get out of it and how much investment I have in it. (Participant 8)

Some participants indicated their health goals were documented which facilitated action, commitment and accountability.

We were setting goals together and I knew that they were written down [in a diary] and there was somebody who was going to be asking me about how I was going with them… Yes, there was a power to that. (Participant 9)

#### 5.1.2 Subtheme 2: Types of Health Goals Co‐Developed

All participants co‐developed a combination of learning‐ and performance‐based health goals with the PHNC. Weight management was a common health goal and goal attainment was achieved by the consumer and PHNC navigating cognitive and practical implementation of physical activity and dietary habits. Participants indicated flexibility to adapt and oscillate between learning and performance‐based health goals according to their capacity and commitment levels.

…having a goal, a weight loss goal, and then figuring out how to get there… If I've got a weight loss goal, then that would be the key to it [diet and exercise]. (Participant 3)

I think goals just change… It's [the goal] not something that has to stay the same…because I'm coming off medication at the moment… that's really impacted on my ability to exercise… I can't exercise when I'm elevated because it makes me more elevated…Realigning my goals and going, okay, well, I can't run four times a week and go to the gym twice a week. Maybe I just run twice a week and go to the gym twice a week. (Participant 8)

Dietary changes involved participants' expressing desires to shift attitudes and habits, indicating their capacity and commitment to these learning and performance‐based health goals. Reported desired dietary changes involved committing to portion control, substitution of unhealthy foods with healthier foods, access to budgeting and resources for groceries and meal preparation.

Portion control was one of the things we were talking about. PHNC recommended why don't you have some celery sticks and carrots. Prepare them and put them in a bag and put them in the fridge and when you feel like a snack, snack on a celery stick or a bit of carrot instead of a bag of chips. (Participant 6)

Desired physical activity changes reported by participants involved combining learning and performance‐based health goals including increasing movement through various activities such as incidental exercise, walking or gardening and establishing an exercise routine.

I've got work. I do manual labour. It's not like hard labour, it's just cleaning. But it gets me moving, which is good. (Participant 5)

Some participants co‐developed learning‐based health goals aiming to reduce or cease their cigarette smoking and/or alcohol consumption.

I'd love to cut down. I can't stand spending all that money on it—it's [cigarettes] $57.00 for a packet. (Participant 4)

Another goal was reducing my alcohol usage which I'm sort of struggling with at the moment. (Participant 7)

Management of comorbid physical illnesses such as diabetes and hypertension were considered indirect health goals by consumers.

My goal was to reduce some weight…to stay on top of my diabetes, which I'm managing… My blood pressure is down, so that's a good benefit of being in the health study and losing the weight. (Participant 12)

### 5.2 Theme 2: Techniques, Barriers and Facilitators to Implementing and Sustaining Health Goals

Participants identified helpful behavioural change techniques used by the PHNC to assist with overcoming barriers and positively implementing and sustaining their health goals.

#### 5.2.1 Subtheme 1: Techniques for Implementing Health Goals

Implementation of weight management health goals reportedly involved learning and performance‐based approaches including attitude and behavioural changes to their diet and physical activity, medication management, use of mobile applications and paper‐based aides, and referral to allied healthcare professionals.

…I'm on a lot of medicines, we talked about managing side effects of the medicines to help with weight loss and things like trying out My Fitness Pal to see if that would help. Just trying different things and see what worked for me…I ended up seeing a dietician. (Participant 13)

Regarding dietary changes, participants reported the PHNC provided verbal dietary advice accompanied by written material to support desired behavioural change. To improve performance, participants recalled the PHNC suggesting mobile applications and paper‐based tools to track their grocery budget and food or caloric intake. These regular participant performance feedback tools helped facilitate portion control, routine meals, reducing food‐related expenses and organise meal preparation.

[After a discussion], PHNC writes down on the paper and gives me a diet to follow and talks to me about foods and my health, my diabetes. I find [PHNC] very helpful. PHNC taught me a lot. (Participant 4)

The PHNC encouraged participants to use tools such as diaries or mobile applications to personalise, plan and monitor physical activities. Receiving performance feedback and monitoring progress of physical activity resulted in participants' feeling accountable and committed to attaining their goals.

I had a diary, and I'd write down what time I left and what time I'd come back. I just kept writing down when I'd go for a ride or a walk, and just kept track of the numbers there. Plus, my PHNC calculated it up how many minutes a fortnight. (Participant 14)

For participants with learning‐based goals to reduce or cease cigarette smoking and/or alcohol consumption, the PHNC suggested harm minimisation strategies. Participants with performance‐based goals, recall being advised to substitute cigarettes with nicotine replacement therapy, implement a ‘drinking’ schedule to reduce alcohol consumption and continue accessing support from the multidisciplinary team.

PHNC tells me if I can try and get patches or a e‐smoke that it'll help me get off them…I'm going to try and get some patches, so I don't smoke through the night. (Participant 4)

…I used to drink every day. So now I've changed to three days a week and I don't drink before five o'clock in the afternoon…. I had been seeing a drug and alcohol counsellor and we had it under control and then I sort of lost control. Then I got it back under control now again and that was with the help of [PHNC] and [drug and alcohol counsellor], and the doctors. (Participant 6)

Comorbid physical illnesses such as diabetes and hypertension were reportedly managed by the PHNC referring participants to appropriate allied healthcare professionals. Additionally, the PHNC applied health coaching to motivate behaviour change such as reducing sugar consumption.

I have Type 2 diabetes… I was referred to the diabetes clinic, which has helped me… I've been drinking meal replacements, I've cut out sugar, I've cut out fats. I'm just trying to lose some weight now… (Participant 5).

#### 5.2.2 Subtheme 2: Barriers

Some participants cited COVID‐19 as a barrier to implementing and sustaining learning and performance‐based health goals. The social distancing rules imposed by the Australian government presumably disrupted participants' momentum to sustain their health behaviour changes. Appointments with the PHNC were converted to telehealth, although some participants preferred in‐person appointments.

…During COVID‐19, I stopped exercising, I got depressed, I was drinking a lot. The PHNC continued to do phone consults with me, even though there couldn't be faced to face consults, and never pressured me into any big changes (Participant 8)

Other participants attributed their reduced capacity for behaviour change to mental ill‐health symptom fluctuation and medication side‐effects. Consequently, these participants experienced low energy and motivation precluding the adoption of physical activity and dietary advice from the PHNC.

…I think I'd plateaued with my weight…It's a number of factors that have all conspired together against me [mental state, sleep apnoea and health issues]. (Participant 9)

Some participants experienced burden and guilt regarding their perceived inability to implement and sustain their performance‐based health goals.

The pressure to meet those requirements is just another burden that adds to the burden. (Participant 2)

Some were hospitalised whilst working with the PHNC. The reported limited food choices in hospital hindered their dietary changes and contributed to subsequent weight gain though excess caloric intake and reduced opportunity for physical activity.

…I was vegan, so that means it was…really crap food in the hospital, which means I was eating a lot of white bread and jam to substitute. (Participant 8).

#### 5.2.3 Subtheme 3: Facilitators

Participants attributed the therapeutic partnership with the PHNC, performance feedback, and positive reinforcement as facilitators to adopting, implementing, and sustaining their health goals. These reflections underscore participants' appreciation of the PHNC's support and validation of their efforts to sustain existing and new health behaviours.

PHNC is encouraging, and you can tell that they're also interested and that they're very sincere when they do talk to you…They got to know you a bit before they asked you questions…What I got out of this is just reinforcement that I'm doing a good thing and I'm okay with it. (Participant 1)

Accountability partnerships nurtured by a foundation of therapeutic partnership, regular contact, performance feedback and health coaching from the PHNC were identified as facilitators to sustaining health goals by participants. Participants perceived being accountable to a supportive person, including their social support system, reinforced behaviour changes and ability to commit to health goals.

… I'd be accountable to someone [PHNC]… So, I'd have someone to bounce ideas off, and who keeps track of my health. Because I hardly go to the doctors myself, but having blood tests every three months, and having my weight measured, and stuff like that, keeps me accountable, at the end of the day. (Participant 12)

Some participants considered the environment created by the PHNC facilitated their intrinsic personal motivation and commitment to implement and sustain health goals.

…Basically, [PHNC] kept me motivated … I just stayed being motivated with it. (Participant 14)

In instances where participants experienced disruptions, the PHNC was perceived to remain accessible, enabling negotiated adjustment of health goals to align with current capacity and commitment levels. Participants indicated the PHNC used health coaching approaches that included adaptable and personalised advice and performance feedback.

The PHNC has got valid advice… You incorporate that advice into your daily and weekly and monthly habits, so that those things can become part of your regime, incorporate it as an indirect outcome. As opposed to [criticism]. That takes quite a bit of thinking and strategising, that's how do you get around the mental [barriers]. (Participant 2)

### 5.3 Theme 3: Impact of Health Goals

Participants considered implementing and sustaining health behaviour changes had a positive impact on other domains of their health such as social functioning and activities of daily living. Attending appointments with the PHNC and other allied healthcare professionals and implementing dietary habit changes increased exposure to social settings and facilitated self‐care.

It's a bit like an outing [appointments], like get out of the house and do something…It's good socialising. (Participant 6).

[Grocery shopping was] getting me out more of my home and just by doing more things around the house like housework and just maintaining myself and when I'm out and about, whatever I may be doing. It got me out a lot more. It got me more active. (Participant 14)

Some participants with comorbid physical health issues such as hypertension and diabetes indicated positive impacts of weight loss strategies to their increased confidence, motivation and commitment to sustain health behaviour changes.

It's [stable BGL] made me a little bit more confident… [PHNC] knows that I'm eating the right foods for my diabetes and by January, February, I should be able to lose a little bit of weight. (Participant 4)

I generally feel more motivated to look after my health. (Participant 12)

Other participants reflected how increased physical activity, and healthier dietary habits improved fitness levels, mobility, breathing, and reduced physical pain.

…I don't feel as heavy. I eat more vegetables, and I know that I can feel my tummy…I can breathe easily… I'm lighter, it felt easy and more good breathing, and able to walk more, able to concentrate more. Now the constipation is gone. So, with constipation there are a lot of problems coming up like pain in the body all the time, and in the shoulder…all those sorts of things have gone now. (Participant 11)

Some participants with learning‐based health goals attributed attitude changes as catalysts for adopting, implementing and sustaining their increased physical activity and healthier dietary habits. These participants perceived positive health attitudes, increased health knowledge and awareness, contributed to behaviours leading to significant weight loss.

I've lost a bit of weight [26kg] … [PHNC] Just made me think that I should do some more exercise or eat more vegetables or less junk food. (Participant 7).

## 6 Discussion

The study explored perceptions and experiences of consumers goal‐setting to co‐develop and implement health goals with the PHNC. Findings suggest collaborative care‐planning processes aligning with goal‐setting theory facilitated co‐development and implementation of varying learning and performance‐based health goals. Types of health goals identified by consumers' included weight management, encompassing physical activity and dietary behaviour changes, and smoking and alcohol reduction or cessation. Consumers experienced barriers such as mental health symptom fluctuation, medication side‐effects, and socioeconomic factors when implementing health goals, underscoring the need for tailored and comprehensive support. Barriers were effectively mitigated by the PHNC who applied elements of goal‐setting theory such as assessing, co‐developing, and adapting health goals congruent with consumers' capacity. Subsequently, consumers reported positive impacts of health goals on their overall health.

Goal‐setting is frequently used to facilitate health behaviour change (Swann et al. 2021). In this study, goal‐setting involved the PHNC working with consumers to co‐develop and implement personalised health goals such as increasing physical activity, changing dietary habits, or reducing cigarette smoking or alcohol consumption. Consumers described an empathetic health coaching approach to goal‐setting whereby the PHNC respected consumers' autonomy and supported alignment of health goals with their physical and mental capacity. Descriptions of goal‐setting by consumers reflect a combination of learning‐and performance‐based health goals (Kettle et al. 2022; Swann et al. 2021). Health goals co‐developed by the consumer and PHNC aligned with core tenets of goal‐setting theory. This involved setting specific and challenging learning‐ or performance‐based goals moderated by participants' ability, commitment, resources and feedback on their performance (Kettle et al. 2022; Swann et al. 2021). Learning‐based goals were favourable for consumers commencing new behaviours or habit shifts as they involve cognitive processes of planning approaches to implementing health goals (Swann et al. 2021; Whatnall et al. 2021). Conversely, performance‐based goals mainly benefited consumers with the ability and commitment to change as they seek to implement activities to achieve intended health goals (Swann et al. 2021). Whilst some consumers in this study reflected high levels of personal motivation, most experienced personal challenges. Considering consumers experience high levels of sedentary behaviours (Bort‐Roig et al. 2019), learning‐based goals may be better suited to prevent negative psychological impacts such as guilt or burden resulting from unattainable goals. Therefore, determining goal types, learning or performance‐based, should be a process guided by person‐centred approaches whereby healthcare professionals working with consumers assess and recognise their physical and mental abilities to achieve goals.

Consumers' ability and commitment to health goals were reduced by barriers such as mental illness symptom fluctuation, negative psychological impacts resulting from performance feedback, and medication side effects resulting in lethargy and amotivation. Additionally, reported financial constraints and COVID‐19 limited consumers access to resources or equipment for physical activity and dietary changes (Roberts et al. 2022). In this study, COVID‐19 may have impacted the participants' adherence to their health goals and outcomes of the PHNC intervention; however, no participants reported negative impacts on their goal setting or perception and experience with the PHNC. Similar barriers of lethargy, amotivation and financial limitations are reported by people diagnosed with chronic illnesses such as diabetes who are supported in primary care settings (Foo et al. 2020; Schmidt et al. 2020) indicating additional challenges to be overcome when co‐developing and implementing health goals with consumers. Reported negative psychological impacts resulting from performance feedback may indicate incongruency between types of co‐developed health goals and consumers' ability and commitment levels (Swann et al. 2021). Taken together, awareness of additional complexities experienced by consumers regarding their physical and mental health is crucial. Early detection and mitigation of these perceived barriers prior to and during implementation is fundamental in guiding and co‐developing health goals.

Mediators for consumers' personal barriers were described as strong therapeutic relationships, access to resources, continuity of care with the PHNC, flexible scheduling, proactive person‐centred approaches, adaptability and positive reinforcement. Viewed broadly, approaches used by the PHNC to mitigate consumer barriers reflect elements from self‐determination theory, motivational interviewing and goal‐setting theory. Self‐determination theory explores intrinsic and extrinsic factors influencing human motivations and behaviour (Ryan and Deci 2023) whilst motivational interviewing involves guiding individuals to recognise and address psychological barriers related to undesirable behaviours (He et al. 2023). Self‐determination theory and motivational interviewing were exemplified by the PHN's approach to supporting consumers with learning‐based health goals. Consumers were supported to establish autonomous motivation and solution‐focused approaches to generate, plan and implement ideas directed towards desired health behaviour changes (Teixeira et al. 2020). Additionally, goal‐setting theory was applicable during the regular contact between consumers and PHNC, which included performance feedback and positive reinforcement. These approaches mitigated barriers and enhanced consumers' commitment to their health goals. Current research indicates a modest effect on behaviour changes from health interventions informed by self‐determination theory (Ntoumanis et al. 2021) however, interventions fostering individual autonomy are strongly related to effective performance (Ryan and Deci 2023). Creating environments that support autonomy and satisfaction stimulates behavioural change (He et al. 2023; Wood et al. 2023; Ryan and Deci 2023). Findings from this study suggest approaches used by the PHNC combined self‐determination, motivational interviewing and goal‐setting theory warrant future research to investigate the effectiveness of combining principles from these theories regarding consumer physical health behaviour changes.

When salient properties to facilitate health goal implementation are present, that is, combining theoretical frameworks and aligning goal types with capacity and commitment levels, consumers report being more inclined to act. Consumers' descriptions of positive impacts to their health outcomes were previously reported (Tabvuma, Stanton, and Happell 2022). To summarise, physical improvements were related to diets, mobility, fitness levels and management of comorbid health issues. Additionally, perceived social functioning improved because of increased participation in community activities and mental health outcomes included consumer satisfaction with goal‐setting, empowerment and improved confidence. Taken together, these perceived improved personal health outcomes parallel a systematic review and recent literature regarding effectiveness of Shared Decision Making (SDM) on patient outcomes (Shay and Lafata 2015; Faiman and Tariman 2019; Hughes et al. 2018). The review and subsequent literature indicated SDM was most likely associated with affect‐cognitive patient health outcomes, medication adherence, behavioural and health outcomes. Even in the absence of clinically significant outcomes resulting from physical health interventions (Ekelund et al. 2019; Kettle et al. 2022; Curtis et al. 2024), consumers report positive personal outcomes (Hughes et al. 2018; Tabvuma, Stanton, Browne, and Happell 2022). Future research may need to combine personal and clinical outcome measures that recognise the additional complexities experienced by consumers.

## 7 Limitations and Strengths

The study sample was limited to English‐speaking consumers diagnosed with psychosis and accessing the community mental health service. The COVID‐19 pandemic may have impacted the accessibility of resources such as exercise facilities, consequently impacting participants' adherence to health goals. However, no participants reported negative impacts on their perception and experience with the PHNC. The nature of exploratory qualitative research designs, the study using a convenience sampling strategy to recruit participants from a single setting in Australia, small sample size and scope of research aim precludes the transferability of findings to all mental health consumers. However, this design is appropriate to gather in‐depth perceptions and experiences about facilitating health behaviour change. Additionally, focusing on this cohort is a strength given the marginalisation of their physical health outcomes. Lastly, to prevent bias and increase rigour, the trustworthiness of the data was enhanced through reflexivity and triangulation.

## 8 Conclusion

Understanding consumer experiences and perceptions with goal‐setting processes facilitated by the PHNC in relation to health behaviour changes provides guidance on effective approaches to co‐develop, implement, and sustain health goals. Importantly, the PHNC assessing and recognising consumers' ability to commit, resource and implement their health goals contributed to positive health behaviour changes. Moreover, consumer perceptions show alignment of PHNC health advice and coaching with various behavioural change theories and adapted learning and performance‐based health goals in response to consumers' abilities and presenting obstacles. Consumers indicated a positive impact on several domains of their health, indicating the value of the PHNC in supporting behaviour change and directing future research regarding consumer physical health interventions to underpin behaviour change theories and measure both clinical and personal outcomes.

## 9 Relevance for Clinical Practice

Despite the increasing application of physical health interventions in practice, consumers continue to experience increased morbidity and mortality compared with the general population. Systemic and personal barriers challenge consumers' abilities, commitment, and resourcing to health behaviour changes. Results of the current study support embedding PHNCs in mental health services to facilitate health behaviour change through goal‐setting. To circumvent personal barriers consumers' experience in relation to adopting health behaviour changes, goal‐setting moderators and selecting appropriate goal types should be strongly considered. Given the limited goal‐setting research in this area, future research is warranted to investigate the translation and application of goal‐setting theory when delivering physical health interventions to consumers in mental health settings.

## Ethics Statement

Ethics approval was gained from ACT Health Human Research and Ethics Committee (approval number 2020/ETH.00081), prior to participant recruitment.

## Consent

Informed consent was obtained from all participants prior to the interview.

## Conflicts of Interest

The authors declare no conflicts of interest.

## Data Availability

The data that support the findings of this study are available on request from the corresponding author. The data are not publicly available due to privacy or ethical restrictions.
